# Regulated cell death joins in atherosclerotic plaque silent progression

**DOI:** 10.1038/s41598-022-06762-y

**Published:** 2022-02-18

**Authors:** Elena Uyy, Viorel I. Suica, Raluca M. Boteanu, Aurel Cerveanu-Hogas, Luminita Ivan, Rune Hansen, Felicia Antohe

**Affiliations:** 1 Department of Proteomics, Institute of Cellular Biology and Pathology “Nicolae Simionescu” of the Romanian Academy, 8, B.P. Hasdeu Street, P.O. Box 35-14, 050568 Bucharest, Romania; 2 Department of Health Research, SINTEF Digital, Trondheim, Norway; 3 Department of Circulation and Medical Imaging, Norwegian University of Science and Technology, Trondheim, Norway

**Keywords:** Diseases, Risk factors, Cellular signalling networks, Data acquisition, Data integration, Data mining, Data processing, Databases, Protein analysis, Biomarkers, Predictive markers, Cell death

## Abstract

Non-apoptotic regulated cell death (ferroptosis and necroptosis) leads to the release of damage-associated molecular patterns (DAMPs), which initiate and perpetuate a non-infectious inflammatory response. We hypothesize that DAMPs and non-apoptotic regulated cell death are critical players of atherosclerotic plaque progression with inadequate response to lipid-lowering treatment. We aimed to uncover the silent mechanisms that govern the existing residual risk of cardiovascular-related mortality in experimental atherosclerosis. Proteomic and genomic approaches were applied on the ascending aorta of hyperlipidemic rabbits and controls with and without lipid-lowering treatment. The hyperlipidemic animals, which presented numerous heterogeneous atherosclerotic lesions, exhibited high concentrations of serum lipids and increased lipid peroxidation oxidative stress markers. The analyses revealed the significant upregulation of DAMPs and proteins implicated in ferroptosis and necroptosis by hyperlipidemia. Some of them did not respond to lipid-lowering treatment. Dysregulation of five proteins involved in non-apoptotic regulated cell death proteins (VDAC1, VDAC3, FTL, TF and PCBP1) and nine associated DAMPs (HSP90AA1, HSP90AB1, ANXA1, LGALS3, HSP90B1, S100A11, FN, CALR, H3-3A) was not corrected by the treatment. These proteins could play a key role in the atherosclerotic silent evolution and may possess an unexplored therapeutic potential. Mass spectrometry data are available via ProteomeXchange with identifier PXD026379.

## Introduction

Thoracic aorta atheroma is a common manifestation of systemic atherosclerosis. Large cohort studies from the last decade demonstrate that plaques from any part of the thoracic aorta represent significant risk factors for a dismal outcome, such as stroke or myocardial infarction, threatening the long-term survival of heart surgery patients^1–3^.

Atherosclerosis is considered a multifactorial disease, and classical treatments based on lipid-lowering or anti-inflammatory strategies have led to a significant reduction of clinical events. However, a considerable residual risk of cardiovascular-related mortality remains.

Cells in the atherosclerotic lesions can die in several ways^4^, and the inflammatory response related to each form of cell death is highly variable. Apoptosis leads to immunologically silent responses, whereas non-apoptotic regulated cell death (e.g.: ferroptosis and necroptosis), which does not involve caspase activation, causes the release of inflammatory signals^4^. Ferroptosis and necroptosis are two recently discovered forms of programmed necrosis involved in various pathologies including atherosclerosis. These necrotic processes lead to disruption of the plasma membrane and release of the cellular contents and various damage-associated molecular patterns (DAMPs)^5^. However, the mechanisms behind their regulation are not very well defined.

Several studies on human patients attest the implication of necroptosis in atherosclerotic lesions^6^. Necroptosis results in the rupture of the plasma membrane and subsequent passive release of the intracellular content into the surrounding microenvironment. The intracellular content consists of various molecules that promote immune mediated pro-inflammatory responses^7,8^.

Ferroptosis is characterized by the production of reactive oxygen species (ROS) from stress induced accumulated iron and lipid peroxidation. This cell death occurs in multiple physiological and pathological processes, such as cancer, neurodegenerative disease, nephropathy and atherosclerosis. The regulated proteins generated during cell death, along with the released DAMPs, accumulate in advanced plaques and generate instability that ultimately leads to plaque rupture and other clinical events that are difficult to predict^5^.

In this regard, we used an in vivo experimental model of atherosclerosis in New Zeeland White rabbits. The animals received either hyperlipidemic diet or standard diet together with a lipid-lowering treatment following a high fat diet to assess specific DAMPs’ and other molecules’ abundance, related with regulated cell death (ferroptosis and necroptosis) in various stages of atherosclerotic plaques development. Ascending aorta fragments were subjected to mass spectrometry-based proteomic analysis and histological assays, and the results were validated by biochemical, immunological and gene expression assays.

Various regulated cell death markers and DAMPs have thus been the focus of our study to reveal their potential association with the different stages of atheroma plaque progression and the residual risk of cardiovascular-related events even after lipid-lowering treatment. The results lead to a better understanding of the non-apoptotic regulated cell death in plaque progression and the future prospects of adequate personalized therapy in clinical practice.

## Results

### Confirmation of the hyperlipidemic animal model

**Figure 1:**
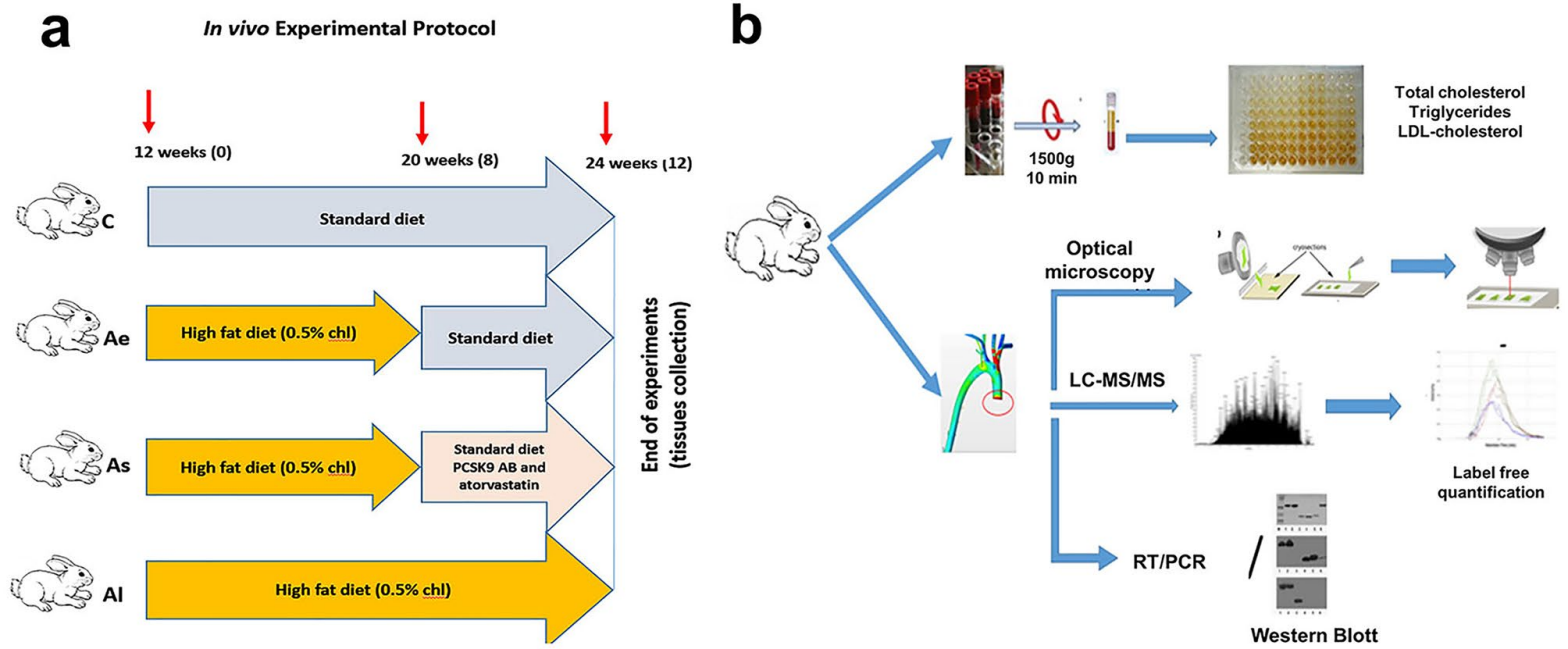
Experimental workflow showing the animal design and methodological approaches. (**a**) Schematic representation of the experimental setup to induce atherosclerotic lesions in the aorta of New Zealand White rabbits: control group (C, n = 7) received standard chow diet for 12 weeks; atherosclerotic group in early stage (Ae, n = 6) which was fed a high fat diet (0.5% cholesterol and 5% corn oil) for the first 8 weeks followed by standard diet for the remaining 4 weeks; a stabilized atherosclerotic group (As, n = 8) which received a high fat diet for 8 weeks, followed by standard food combined with lipid-lowering treatment for the last four weeks; the late atherosclerotic group (Al, n = 8) that received a high fat diet for 12 weeks. (**b**) Workflow of combined approaches followed to study atherosclerosis in aortic tissue and blood samples. Adequate sample preparation was achieved for high-performance nano-chromatographic tandem mass spectrometric, histological, biochemical, immunological and gene expression analysis.

The experimental design and the methodological approaches were illustrated in the following graphical representation, described in detail in the materials and methods section (Fig. 1a, b).

**Figure 2:**
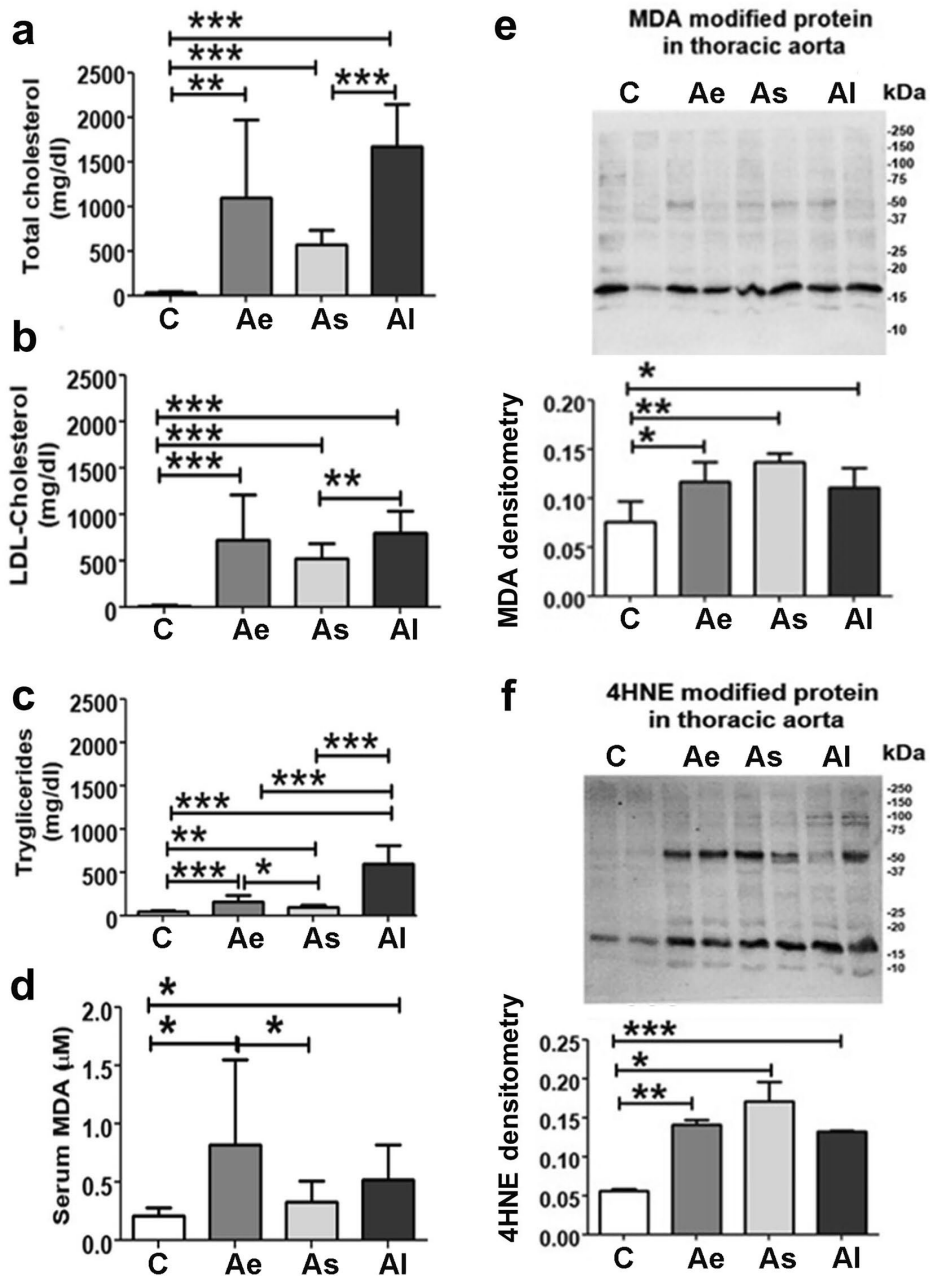
Biochemical lipid assays in sera and Western blot for malondialdehyde- (MDA) and 4-hydroxynonenal- (4HNE) protein level in the ascending aorta’s homogenate. Sera quantitation of (**a**) total cholesterol, (**b**) LDL-cholesterol, (**c**) triglycerides and (**d**) MDA levels for all animal groups included in the study after twelve weeks. Tissue homogenate Western blotting and associated densitometric analysis showed changes in (**e**) MDA- and (**f**) HNE-protein level in the four experimental groups. Ponceau S staining was used for normalisation of the proteins’ levels. C: control group (n = 7), Ae: early atherosclerotic group (n = 6), As: stabilized atherosclerotic group by lipid-lowering treatment (n = 8) and Al: late atherosclerotic group (n = 8). Data are expressed as means ± standard deviation (SD); *p˂0.05; **p˂0.01; *** *p*˂0.001. Full images of Western Blot membranes included in this figure can be found in Supplementary S1.

At the end of the experiment, blood was collected from the auricular vein of all animals and the sera were subsequently used in the biochemical assays (Fig. 2a–d).

The hyperlipidemic diet induced a significant increase (*p* ≤ 0.01) of serum lipid parameters (total cholesterol, triglycerides, LDL-cholesterol) collected from Ae, As and Al when compared to the healthy animal group, C. The total cholesterol values were higher by ~ 32.1-fold (*p* ≤ 0.01) in Ae; by ~ 16.8-fold (*p* ≤ 0.001) in As and by ~ 49.28-fold (*p* ≤ 0.001) in Al rabbits versus the control group (Fig. 2a). Moreover, the LDL-cholesterol value showed a ~ 55.5-fold increase in Ae (*p* ≤ 0.001), a ~ 39.88-fold increase in As (*p* ≤ 0.001) and a ~ 61.37-fold increase in Al (*p* ≤ 0.001) when compared to serum level in control groups (Fig. 2b). The triglyceride values assayed in Ae serum were higher by ~ 3.38-fold (*p* ≤ 0.001); in As by ~ 2.1 fold (*p* ≤ 0.01) and in Al by ~ 13.1 fold (*p* ≤ 0.001) versus control samples (Fig. 2c).

Switching to a standard diet after two months and starting a lipid-lowering treatment (in the As group) induced a decrease in total cholesterol by ~ 2.93-fold (*p* ≤ 0.001), LDL-cholesterol by ~ 1.54-fold (*p* ≤ 0.01) and triglycerides by ~ 6.21-fold (*p* ≤ 0.001) compared to the Al group (Fig. 2a–c). Notably, a ~ 3.87-fold (*p* ≤ 0.001) lower level of serum triglycerides was observed once the hyperlipidemic diet was switched to a standard one (Ae vs Al groups) (Fig. 2c).

### Hyperlipidemic diet induced lipid peroxidation oxidative stress

A major consequence of increased oxidative stress was the peroxidation of membrane lipids in the lesion areas. Malondialdehyde (MDA) has been used as a convenient biomarker for lipid peroxidation of fatty acids due to its reaction with thiobarbituric acid (TBA) measured by the resulting red colored product^9^. Our study reveals that atherosclerosis induced the serum overproduction of lipid peroxidation products MDA by ~ 3.9-fold (*p* ≤ 0.05) in the Ae group and by ~ 2.5-fold (*p* ≤ 0.05) in the Al compared with the C group. Notably, lipid-lowering treatment decreases serum MDA concentration by ~ 2.5-fold (*p* ≤ 0.05) in As vs Ae group (Fig. 2d).

The main products of lipid peroxidation, MDA and hydroxynonenal (HNE), oxidize proteins altering their structure, activity, and physical properties^10^. HNE- and MDA-proteins possess robust pro-inflammatory properties, constituting a novel class of DAMPs generated during high oxidative stress. In our analysis, we identified more MDA-modified proteins when comparing the proteome isolated from Ae (~ 1.54-fold and *p* < 0.05), As (~ 1.8-fold and *p* < 0.01) and Al (~ 1.46-fold and *p* < 0.05) to the healthy animals (Fig. 2e, f). Additionally, we demonstrated increased levels of protein carriers of HNE as a response to hyperlipidemic stress in ascending aortas isolated from Ae (~ 2.5-fold and *p* < 0.01), As (~ 3-fold and *p* < 0.05) and Al (~ 5.6-fold and *p* < 0.001) animals. Interestingly, lipid-lowering treatment induced a decrease in serum MDA concentration (Fig. 2d) but with no significant effect on HNE- and MDA-modified proteins in the aortic tissue (Fig. 2e, f).

### Atherosclerotic lesions heterogeneity is correlated with appearance of iron deposits

**Figure 3:**
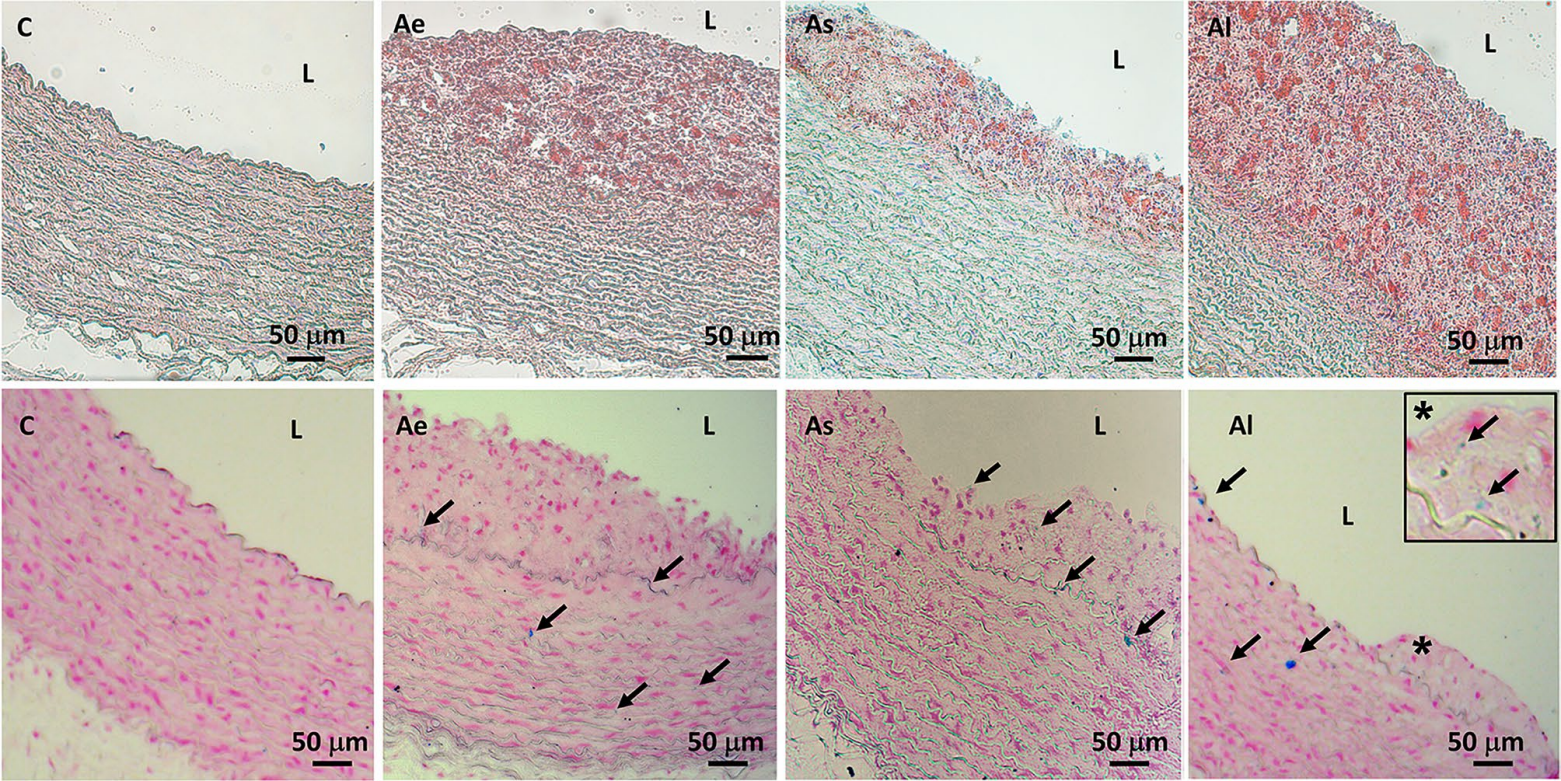
Representative histopathological staining of the ascending aortic segments, embedded in OCT and frozen in liquid nitrogen**.** The upper panel was obtained after double staining with Oil Red O and Hematoxylin of the serial sections, for lipid deposits’ detection. The lower panel sections were stained with Prussian Blue and Nuclear Fast Red solutions to evidence ferrous and ferric iron in blue, the cytoplasm in pink and cell nuclei in red. C: control group, Ae: early atherosclerotic group, As: statin and PCSK9 antibody stabilized atherosclerotic group and Al: late atherosclerotic group (see also the inset for higher magnification, × 40).

The development of atherosclerotic lesions and lipid deposits were highlighted by parallel histological staining with Oil Red O and Hematoxylin of ascending aorta cryosections (Fig. 3).

All rabbits fed a hyperlipidemic diet (for two months in As and Ae or for three months in Al group) showed well developed heterogeneous atherosclerotic plaques compared to the control group C. The presence of iron deposits, a determining factor in the development of the ferroptosis process^11^, was highlighted by the Prussian Blue and Nuclear Fast Red staining. Iron deposits were predominantly found inside the internal tunica of the ascending aorta, in the subendothelial space and in the lesions harvested from the ascending aorta of the Al rabbits. The aortic control sections (C) demonstrated a lack of iron deposits evidenced by the absence of Prussian Blue staining (Fig. 3, lower panel).

### Alteration of DAMPs and regulated cell death pathways (ferroptosis and necroptosis) in ascending aorta and THP-1 monocytic cell line under hyperlipidemic stress

**Figure 4:**
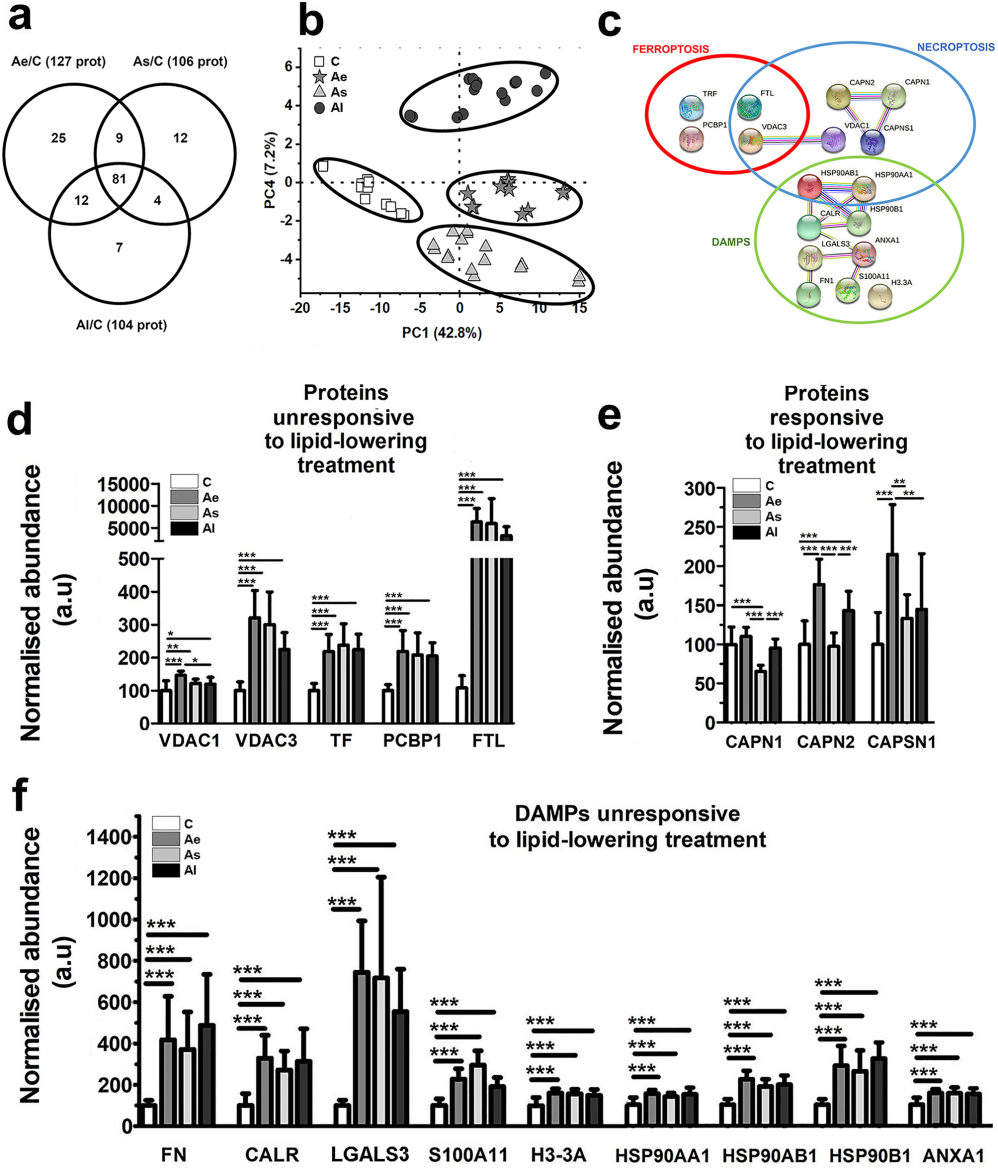
The global proteomic analysis of the aortic homogenates extracted from all animals included in this study. (**a**) Venn diagram revealing the number of differentially abundant proteins over the control in Ae, As, and Al atherosclerotic groups. All sets of experiments were performed in technical duplicates. (**b**) Mass spectrometry data of the differentially abundant proteins from aortic homogenate extracts analyzed by PCA plots showing the spatially disparate protein profiles of Ae, As, Al atherosclerotic groups compared to control, C. (**c**) Protein interaction networks identified in differential proteomes of aortic tissue from all animals correlated with regulated death KEGG pathways (ferroptosis and necroptosis) and DAMPs' associated release as predicted by STRING bioinformatic tool. Nodes represent individual proteins belonging to cell death pathways (ferroptosis and necroptosis) and to the DAMPs associated proteins identified by mass spectrometry (encircled in red, blue and green, respectively). Node annotations in clusters mean: VDAC1 (voltage-dependent anion-selective channel protein 1), VDAC3 (Voltage-dependent anion-selective channel protein 3), TF (serotransferrin), PCBP1 (poly (RC) -binding protein 1), FTL (Ferritin light chain), CAPSN1 (Calpain small subunit 1), CAPN2 (Calpain-2 subunit catalytic), CAPN1 (Calpain-1 catalytic subunit), CAPSN1 (Calpain small subunit 1), HSP90AA1 (Heat shock protein HSP 90-alpha), HSP90AB1 (Heat shock protein HSP 90-beta), ANXA1 (annexin A1), LGALS3 (galectin 3), HSP90B1 (endoplasmin), S100A11 (protein S100A11), FN (fibronectin), CALR (calreticulin), H3-3A (histone H3.3). The normalized abundance changes of the identified proteins induced by hyperlipidemia implicated in KEGG inter-relation map of ferroptosis and necroptosis pathways. Proteins are divided into two groups: (**d**) proteins unresponsive to lipid-lowering treatment: VDAC1, VDAC3, TF, PCBP1, FTL and (**e**) proteins responsive to lipid-lowering treatment: CAPN1, CAPN2, CAPSN1. (**f**) Histograms showing the differentially abundant DAMPs quantified in the ascending aorta samples of C, Ae, As and Al groups, using label-free relative mass spectrometric analysis: FN, CALR, LGALS3, S100A11, H3-3A, HSP90AA1, HSP90AB, HSP90B1, ANXA1. C: control group (n = 7), Ae: early atherosclerotic group (n = 6), As: stabilized atherosclerotic group with lower lipid treatment (n = 8), Al: late atherosclerotic group (n = 8). Data represent the mean ± SD; *p < 0.05; **p < 0.01; ***p < 0.001).

Tissue homogenates from ascending thoracic artery were used to identify the significantly altered protein levels in the lesion-prone areas. The shotgun proteomic analysis revealed a high number of molecules that were significantly altered (*p*-value adjusted using Benjamini–Hochberg correction for False Discovery Rate) by the high-fat diet (Ae/C and Al/C) or after the hypolipidemic treatment vs control (As/C). Thus, atherosclerosis induced the spectral abundances alteration (at least 1.5-fold, *p* < 0.05) of 127 proteins after hyperlipidemic diet administration for two months (Ae/C) and 104 proteins after the hyperlipidemic diet administered for three months (Al/C). The hyperlipidemic diet associated with lipid-lowering medication induced an abundance change detected (at least 1.5-fold, *p* < 0.05) for 106 proteins when As samples were normalized to C, as shown in Fig. 4a. The results of the PCA analysis (Fig. 4b) indicate that the quantitative changes of the proteins after the hyperlipidemic diet with or without lipid-lowering treatment administration were particularly specific. The spatial clear separation highlights a high potential of our bioinformatics analysis to identify biomarkers relevant to residual atherosclerotic risks under lipid-lowering therapy. The statistically regulated proteins (> 1.5 fold, *p* ≤ 0.05) by hyperlipidemia with or without the low fat therapy were correlated with the KEGG (Kyoto Encyclopedia of Genes and Genomes) databases^12^ using the STRING program (v 11.2) (Supplementary Table S2). The over-representation (FDR *p*-value ≤ 0.0335) of the ferroptosis and necroptosis pathways were clearly evidenced as two processes of programmed cell death associated with DAMP release (Fig. 4c).

All seventeen differentially abundant proteins highlighted in Ae, As, and Al atherosclerotic groups (fold change at least 1.5, *p* ≤ 0.05 over control) were correlated with programmed cell death (ferroptosis and necroptosis) and selected DAMPs (listed in Table 1). Notably, these proteins were identified and quantified with high bioinformatics confidence (Sequest HT score > 168 and > 6 unique peptides/protein).

**Table 1:** The list of differentially abundant proteins in the ascending aorta samples from all experimental animals using label-free relative mass spectrometric analysis involved in ferroptosis, necroptosis and associated DAMPs with altered mass spectrometric abundance > 1.5-fold (*p* < 0.05) due to the hyperlipidemic diet administration with or without lipid-lowering treatment.

No.	UniProt accession code	Protein name	Role/implication	Unique peptides	Sequest HT score
1	P51662	Annexin A1	DAMP	52	11,088.35
2	P06813	Calpain small subunit 1	Necroptosis	19	2271.85
3	P06815	Calpain-1 catalytic subunit	Necroptosis	17	546.6
4	P06814	Calpain-2 catalytic subunit	Necroptosis	32	2444.21
5	P15253	Calreticulin	DAMP	40	4956.49
6	O18750	Endoplasmin	DAMP	46	5273.51
7	P09451	Ferritin light chain	Ferroptosis necroptosis	12	785.92
8	Q28749	Fibronectin	DAMP	9	1397.53
9	P47845	Galectin-3	DAMP	15	2059.51
10	P30946	Heat shock protein HSP 90-alpha	Necroptosis DAMP	40	7902.81
11	P30947	Heat shock protein HSP 90-beta	Necroptosis DAMP	39	5443.04
12	P84246	Histone H3.3	DAMP	13	1558.89
13	O19048	Poly(RC)-binding protein 1	Ferroptosis	13	1668.83
14	P24480	Protein S100A11	DAMP	13	3347.37
15	P19134	Serotransferrin	Ferroptosis DAMP	82	16,465.36
16	Q9TT15	Voltage-dependent anion-selective channel protein 1	Ferroptosis necroptosis	16	1907.77
17	Q9TT13	Voltage-dependent anion-selective channel protein 3	Ferroptosis necroptosis	6	168.07

The control group (C), early atherosclerotic group (Ae), atherosclerotic group stabilized by statin and anti-PCSK9 administration (As) and late atherosclerotic group (Al). Parameters of protein inference are also presented (UniProt accession code, name, the number of unique peptides/proteins based on which the protein was identified and Sequest HT protein identification score).

Figure 4d shows that the proteins involved in the ferroptosis signaling pathway are significantly upregulated in all normalized samples from animals that developed atherosclerotic lesions (Ae, As and Al) when compared to controls. These are: voltage-dependent anion-selective channel protein 1 (VDAC1; > 1.2-fold, *p* ≤ 0.05), voltage-dependent anion-selective channel protein 3 (VDAC 3; > 2.2-fold, *p* ≤ 0.001), serotransferrin (TF; > 2.3-fold, *p* ≤ 0.001), poly (RC) -binding protein 1 (PCBP1; by > 2.1-fold, *p* ≤ 0.001) and ferritin light chain (FTL; > 32-fold, *p* ≤ 0.05).

In addition, in all three groups, the plaques revealed iron-positive stained deposits as well, characteristic for oxidative processes. Ferritin, a protein whose function is to act as iron reservoir of the body and is used as a marker in clinical evaluations for iron overload^13^, was found by mass spectrometry relative quantification to be significantly elevated in atherosclerosis plaques, indicating oxidative stress and, eventually, cell death that was triggered by free iron radicals.

The lipid-lowering therapy concomitant with standard diet administration did not influence the ferroptosis proteome, meaning that cells continue to die and release DAMPs that generate an activated inflammatory microenvironment. Being one of the persistent effects of atherosclerosis even under the lipid-lowering treatment, we may count it as one of the interesting residual risks to be evaluated in patients in which the currently used medication and the assumed lifestyle changes have little effect.

In addition, Fig. 4e shows the three proteins belonging to the calpains family that do respond to the lipid-lowering treatment when reporting Ae to As abundance: Calpain-1 catalytic subunit (CAPN1, ~ 1.7 fold and *p* < 0.001), Calpain-2 catalytic subunit (CAPN2, ~ 1.8 fold and *p* < 0.001) and Calpain small subunit 1 (CAPSN1, ~ 1.6 fold and *p* < 0.01) that are involved mainly in necroptosis, the second type of regulated cell death detected under the hyperlipidemic stress.

Also, in our experimental setting, CAPSN1 (Ae/C ~ 2.14 and *p* < 0.001) and CAPN2 (Ae/C ~ 1.76- and *p* < 0.001) were upregulated in early stages after only two months of hyperlipidemic diet. An extended hyperlipidemic diet (administrated to Al group) induced an up-regulation of CAPN2 (Al/C ~ 1.4-fold and *p* < 0.001) protein expression but to a lesser extent compared to the Ae group (Ae/C ~ 1.8-fold and *p* < 0.01) (Fig. 4e).

Unlike immunologically silent apoptosis, ferroptosis and necroptosis are immunogenic^14^. In this regard, using our experimental approach, we detected DAMP molecules in the aortic atherosclerotic tissue, which amplify cell death and promote several inflammatory reactions. In our case, the immunogenic effect of ferroptosis and necroptosis can be correlated with the significant changes in the abundance of the nine selected DAMPs presented in Fig. 4f: FN (fibronectin), CALR (calreticulin), LGALS 3 (galectin 3), S100A11 (protein S100A11), H3-3A (histone H3.3), HSP90AA1, HSP90AB, HSP90B1 (endoplasmin), ANXA1 (annexin A1), that accompany the progression of immune mediated inflammation and vulnerability of atherosclerotic plaques.

The hyperlipidemic diet, independent of the lipid-lowering treatment administration, caused an increase in the abundance of all DAMPs (*p* < 0.001) in the aortic homogenates prepared from Ae, As and Al versus C mentioned in the Fig. 4f. These changes correlate very well with the histological results that revealed extensive atherosclerotic lesions, with increased serum lipid parameters that highlighted the hyperlipidemic status of the animals and with the associated changes of regulated cell death components of the processes (ferroptosis and necroptosis) evidenced by high resolution proteomic analysis.

In addition to proteins involved in ferroptosis, the altered abundances of the already mentioned DAMPs were one of the persistent effects accompanying hyperlipidemia that maintained the residual risk of cardiovascular fatal events, under lipid-lowering therapy.

**Figure 5:**
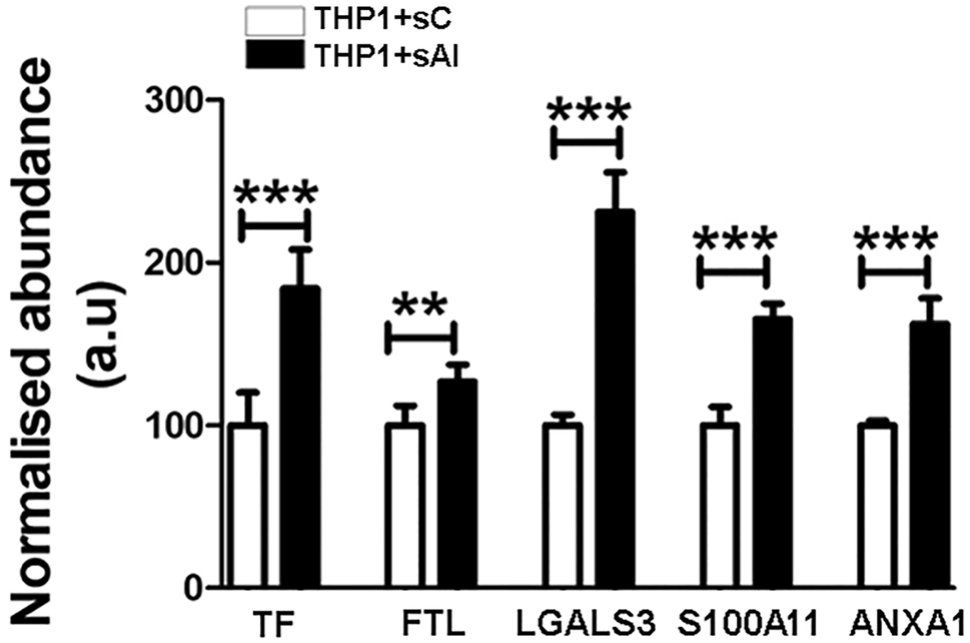
The normalized abundance changes of proteins induced by hyperlipidemic serum in THP-1 cells correlated with iron metabolism and DAMPs**.** Histogram highlighting the normalized abundance changes induced by hyperlipidemia in THP-1 cells exposed to normo-lipidemic serum (sC) or hyperlipidemic serum (sAl). TF and FTL are implicated in iron homeostasis, while LGALS3, S100A11 and ANXA1 are differentially abundant potential DAMPs. Data represent the mean ± SD; **p < 0.01;***p < 0.001). TF: serotransferrin; FTL: Ferritin light chain; LGALS3: galectin 3, S100A11: protein S100A11 and ANXA1: annexin A1.

Recent data sustained that lipid loading begins even in circulating monocytes, which develop a foamy monocyte phenotype and migrate into the arterial wall^15^. Our proteomic results obtained after an in vitro study (Fig. 5), show that the hyperlipidemic serum sAl (harvested from the hyperlipidemic Al animals) caused in the THP-1 human cells an increase in the abundance (*p* < 0.001) of two proteins implicated in iron homeostasis and three potential DAMPs when compared with the control sC (harvested from control animals) These are: serotransferrin (~ 1.8-fold, *p* ≤ 0.001), ferritin light chain (~ 1.3-fold, *p* ≤ 0.01), galectin 3 (~ 2.3-fold, *p* ≤ 0.001), protein S100A11 (~ 1.65-fold, *p* ≤ 0.001) and annexin A1 (~ 1.65-fold, *p* ≤ 0.001). The increased protein levels of galectin-3 and annexin A1 could be construed also as indicators of monocyte-to-macrophage differentiation. The total number of changed proteins were listed in Supplementary Table S3. The results are in correlation with our hypothesis and bring further confirmation of the affected proteins in atherosclerosis but this time by activated cells that trigger the non-apoptotic cell death and selected DAMPs overexpression.

### Validation of mass spectrometry results by alternative methods

**Figure 6:**
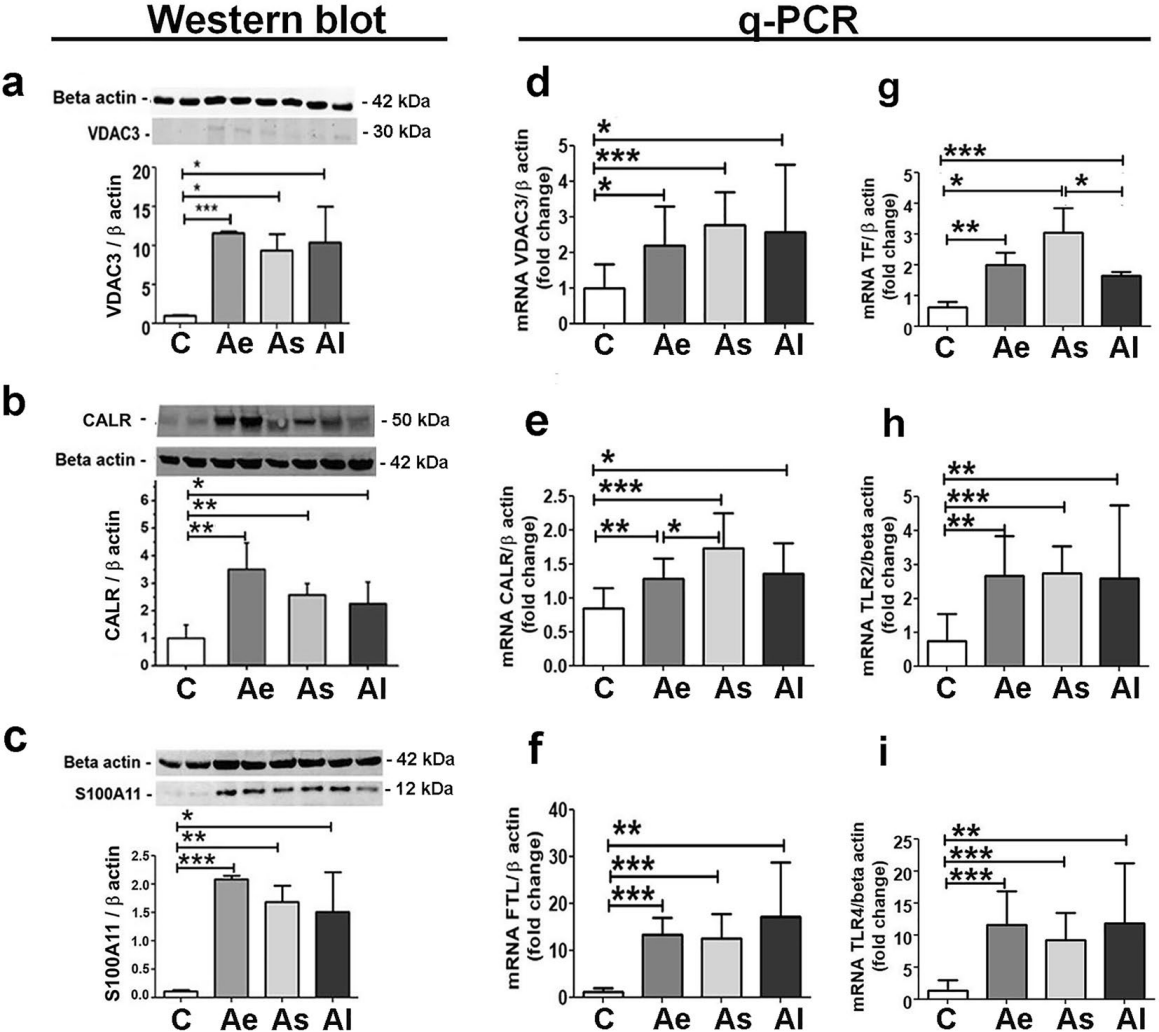
Validation of mass spectrometry data by Western blot and quantitative qPCR methods. The Western blot immune detection and associated densitometry for selected protein markers: (**a**) VDAC3, (**b**) CALR and (**c**) S100A11 and mRNA gene expression level for: (**d**) VDAC3, (**e**) CALR, (**f**) FTL, (**g**) TF, (**h**) TLR2 and (**i**) TLR4 in aortic homogenate prepared from C, Ae, As and Al groups normalized to beta actin protein and gene expression, respectively. C: Control group, Ae: early atherosclerotic group, As: statin and PCSK9 antibody stabilized atherosclerotic group and Al: late atherosclerotic group. Protein and gene levels found for the selected markers are in good corroboration with the mass spectrometry results. Statistical analysis was performed using t Student’s test and results show mean ± SD (*p < 0.05; **p < 0.01 and ***p < 0.001; n > 4). VDAC3: voltage-dependent anion-selective channel protein three, CALR: calreticulin, S100A11: protein S100A11, FTL: ferritin light chain, TF: transferrin, TLR2: toll like receptor 2 and TLR4: toll like receptor 4. The original blots images of the membranes from this figure can be found in Supplementary S1.

The mass spectrometry data were further validated by alternative methods as shown in Fig. 6. Notably, significant increases in VDAC3 (> 5-fold and *p* < 0.05), CALR (> 2-fold and *p* < 0.05) and S100A11 (> 1.5-fold and *p* < 0.05) protein levels were confirmed by Western blotting in homogenates of atherosclerotic tissue extracts from (Ae, As and also Al) compared to aortic control samples (C), (Fig. 6a–c).

Next, gene expression assays were performed to verify the molecules involved in the ferroptosis pathway and the associated released DAMPs (Fig. 6d–i). The gene expression analysis demonstrated significant over-expression of VDAC3 (> 2.2-fold and *p* < 0.05), FTL (> 10.7-fold and *p* < 0.01), TF (> 2.7-fold and *p* < 0.05), CALR (> 1.5-fold and *p* < 0.05), TLR2 (> 3.4-fold and *p* < 0.01) and TLR4 (> 7-fold and *p* < 0.01) mRNA levels in the atherosclerotic ascending aorta compared with healthy aorta samples.

The data presented in this paper corroborate well with previously published results. Thus, statins and PCSK9 inhibitors are mainly used as cholesterol-lowering drugs, but they also have immunoregulatory and anti-inflammatory properties. For some patients the above-mentioned therapies are inefficient and therefore residual cardiovascular risk is maintained^14^. A deeper investigation of how the proteome is affected during or after a lipid targeted treatment can lead to a better understanding of response to lipid-lowering drugs, and ultimately to a more personalized approach in order to reduce the clinical events triggered by atherosclerotic plaque instability.

## Discussions

The major findings of this study bring clear evidence that diet changes and treatment with lipid-lowering drugs (e.g., statins and PCSK9 inhibitors) are not sufficient to stop/ reverse entirely the risk factors for cardiovascular disease in atherosclerosis.

Here we described two important pathological mechanisms by which vascular regulated cell death promotes and amplifies atherosclerosis. The DAMPs' controlled release maintains the inflammatory plaques, leading to potential rupture and vascular accidents. Independent of the lipid-lowering therapy, the increase of DAMPs' abundance (*p* < 0.001) in the aortic wall promotes an oxidative stress environment, confirmed by the accumulated malondialdehyde (MDA) and 4-hydroxynonenal (HNE) modified proteins, and likely contributing to increased atherosclerosis and plaque development.

In the described rabbit animal model, the persistent hyperlipidemic diet induced the upregulation of more than four proteins involved in ferroptosis and altered the abundance of 10 proteins involved in necroptosis (two of them common to ferroptosis, too), known as inflammatory forms of regulated necrotic cell death. Regulated cell death is an important characteristic of advanced atherosclerotic plaques with a major impact on plaque destabilization^16,17^. Growing evidence indicates that pharmacological targeting of these types of death might be a promising approach to stabilize vulnerable plaques and may contribute to the beneficial effects of plaque stabilizing therapies in the future^6,18,19^.

Rabbit was the first and one of the best animal models for the study of lipoprotein metabolism and atherosclerosis^20^. They are sensitive to dietary cholesterol and rapidly develop severe hypercholesterolemia leading to prominent aortic lesions, evidenced also by our experiments. To our knowledge, this is the first study to demonstrate with highly statistical relevance the proteome alteration involved in necroptosis and ferroptosis pathways using a rabbit atherosclerotic model. Although the lesions that form within the ascending aorta of rabbits may be markedly different in their composition/structure compared to clinically-relevant human plaques, the demonstrated biological processes of dying cells within the plaques were also demonstrated previously to be associated with atherosclerosis in small rodents and in vitro cell cultures^21–23^.

We noticed that the therapy with statin and PCSK9 inhibitor, combined with standard diet (as in As group) induced indeed a significant reduction (about 37%) of the MDA and lipid serum concentration supporting the concept of active biological mechanisms that do respond to current lipid-lowering treatment. Among the aldehydes produced by lipid peroxidation, MDA- and HNE- adducts are able to oxidize DNA, proteins, and lipids, altering their structure, activity, and physical properties^9^. They accumulate progressively in the vascular system, leading to cellular dysfunctions and tissue damaging effects^23^. This hypothesis was confirmed by our immunological analysis that revealed an enhanced number of proteins that present MDA and HNE adduct in all three groups (Ae, As and Al) that presented atherosclerotic lesions (Fig. 2e, f). Higher levels of serum MDA concentrations, an end-product of lipid peroxides, may replace lipid peroxides as a biomarker of plaques instability evaluation as previously suggested as well^18^.

Lipid peroxidation also contributes to regulated cell death and recent studies identified lipid peroxidation as the primary driver of ferroptosis^10^. Our novel results indicate that necroptosis and ferroptosis are the most important over represented signaling pathways from all types of cell death forms in the advanced aortic atherosclerotic lesions that do not respond adequately to lipid-lowering treatment with atorvastatin and PCSK9 antibody. The high fat diet induces an abundance alteration of 12 proteins involved in programmed cell death processes (4 proteins in ferroptosis and 10 proteins in necroptosis with two of them common to the two processes, showing clear interconnections). Necroptosis typically occurs when apoptotic cells are insufficiently cleared by neighboring cells via a phagocytic process (efferocytosis). This form of cell death is active in advanced and unstable atherosclerotic lesions in humans as well^6,14^.

Ferroptosis is a type of regulated cell death that is mainly mediated by iron-dependent lipid peroxidation. It has been shown that ferroptosis inhibitor, ferrostatin-1, partially inhibits iron accumulation, lipid peroxidation and alleviate atherosclerosis lesions in the high-fat diet fed ApoE^−/−^mice^20^. In our experiments, all four proteins that correlated with the ferroptosis process (TF, FTL, PCBP1 and VDAC3) showed an increased abundance induced by the hyperlipidemic diet and were not significantly changed by the lipid-lowering treatment (Fig. 4d, e). Also, at gene level, three of them (TF, FTL and VDAC3) presented the same overexpression trend in the aortic homogenates, corroborating with the already mentioned protein increased level.

Serotransferrin (TF) is a serum factor identified as the inducer of ferroptosis which is also implicated in stimulating cell proliferation^24^. Our in vivo and in vitro experimental data revealed an increase of serotransferrin protein and gene expression induced in advanced atherosclerotic plaques and also in ferritin light chain (FTL) iron stores in a soluble, non-toxic, readily available form to be delivered to cells. Important for iron homeostasis, iron is taken up in the ferrous form and deposited as ferric hydroxides after oxidation. In this study, the increase in gene and protein expression of TF and FTL in atherosclerotic lesions as demonstrated by LC–MS/MS and qPCR, was accompanied by the raised iron deposition as shown by Prussian blue reaction (Figs. 3, lower panel, 4d and 6g, f). These results support the idea that ferritin synthesis is regulated by intracellular iron at both the transcriptional and translational levels, while the specific staining revealed the presence of iron deposits mainly in advanced lesions but only in small amount in early human or rabbit lesions as published previously as well^25–27^. Apparently, the paradoxical roles of ferritin involved in atherogenesis largely depends on the stage of atherosclerotic lesion development and the state of iron loading in ferritin macromolecules.

PCBP1 is involved in iron storage pathways, whereas PCPB2 is a regulator of iron transport and recycling^28^. Both PCBP1 and PCBP2 deliver iron to ferritin, although only PCBP1 is involved in the nuclear receptor coactivator 4 (NCOA4)-mediated iron recycling pathway during autophagy^29^. This data is in good agreement with our LC–MS/MS results which demonstrated that the hyperlipidemic diet with or without lipid-lowering treatment led to an over-regulation of PCBP1. The increase in iron storage rather than transfer and recycling, resulted in abundant punctate iron positive deposits (shown by Perl’s Prussian blue staining in Fig. 3, lower panel), revealed predominantly within subendothelial space of intima and tunica media of ascending aorta in high fat fed rabbit.

We also noticed the positive correlation between the abundance of MDA- and HNE- modified proteins in all atherosclerotic groups and the increased protein expression of VDAC3 and VDAC1, that form channels in the mitochondrial outer membrane, with role in the regulation of ATP transport and Ca^2+^ homeostasis^30,31^. These dysregulated mechanisms were well known features of the advanced vascular atherosclerotic lesions.

Calpains, which are calcium-activated proteases, were proposed as novel molecular targets for control the progress of atherosclerosis. High levels of calpains lead to the infiltration of monocytes/macrophages and plasma lipids into the intimal spaces, so an anti-calpain therapy could be beneficial in ameliorating the disease^32^. In our experimental model, administration of lipid-lowering treatment (statins and PCSK9 inhibitor) induced a decrease in abundance for the three members of the calpains family involved in necroptosis: CAPN2, CAPN1 and CAPSN1 (in As vs Ae animals), while the hyperlipidemic diet (in Ae vs C animals) led to higher levels of CAPSN1 and CAPN2. It was speculated that calpains also mediate oxidized LDL-induced apoptotic death in endothelial cells and in monocytes/macrophages can induce proteolytic degradation of ATP-binding cassette transporter A1 (ABCA1) and G1 (ABCG1), which results in impaired cholesterol efflux and subsequent macrophage foam cell formation.

The process of progressive installation of calpain-mediated necroptosis may explain the mass spectrometry results which revealed that 2 months of hyperlipidemic diet (as in Ae animals) induced an increase of CAPSN1 and CAPN2 protein level compared to the extended hyperlipidemic diet (as in Al rabbits). However, the identification of the mechanism of dominant cell death during the progression of atherosclerosis under the control of the calpain systems remains a challenge. Subtype-selective anti-calpain therapy may be highly desirable in order to achieve enhanced understanding of disease mechanisms and improve therapeutic outcomes.

Ferroptosis and necroptosis, less characterized types of regulated necrosis, lead to disruption of plasma membrane and release of the cellular contents and various DAMPs into the extracellular environment. Necrotic cells do not only contribute to the formation and enlargement of the necrotic core, they also are a source of pro-inflammatory DAMPs^33^. Their release and interaction with corresponding TLR molecules (such as TLR2 and TLR4) cause the rapid induction of proinflammatory intracellular signaling cascades and promotes inflammation in the plaque, thereby causing plaque instability.

The immunogenic effect of ferroptosis and necroptosis can be correlated with the notable modification of aortic abundance of the following DAMPs: ANXA1 (annexin A1), LGALS3 (galectin 3), HSP90B1 (endoplasmin), S100A11 (protein S100A11), FN (fibronectin), CALR (calreticulin), H3-3A (histone H3.3), HSP90AA1 and HSP90AB1, induced by the onset of atherosclerotic pathology (Fig. 4f). Administration of a hyperlipidemic diet, independent of the lipid-lowering treatment increased DAMPs abundance in the aortic homogenate of As, Ae and Al (i.e.: ANXA1, LGALS3, HSP90B1, S100A11, FN, CALR, H3-3A, HSP90AA1 and HSP90AB1). These changes correlate with the histological results that showed extensive atherosclerotic lesions, with the sera biochemical assays that highlight the hyperlipidemic characteristics of the animals and with the alterations of regulated cell death mechanisms. In addition to proteins involved in ferroptosis, abundance alteration of other nine DAMPs (ANXA1, LGALS3, HSP90B1, S100A11, FN, CALR, H3-3A, HSP90AA1 and HSP90AB1) is one of the persistent effects of our atherosclerotic setup. We hypothesize that these alterations may play a role in the atherosclerotic residual risk. Follow-up clinical studies are needed to investigate their potential as evolution molecular markers in cases where the prescribed medication doesn’t lead to the desired effect.

Investigations of TF, FTL, PCBP1 and VDAC3 and their different specificities in terms of their roles in iron metabolism, independent of lipid-lowering treatment actions, may also be of high interest in the treatment of iron overload and deficiency. To the best of our knowledge, these results are the first to link impairment of iron homeostasis (TF, FTL, PCBP1) with deregulated mitochondrial membrane proteins (VDAC1, VDAC3) and increased release of specific DAMPs, even under a hypolipidemic treatment and reversal of atherogenic diet.

One important limitation of our study refers to the inability of the applied methodological assays to distinguish between immunologically active DAMPs, which are associated with the extracellular space, and their intracellular homologues which mostly exhibit non-immunological functions. Our results are based on the differential analysis of tissue homogenates, so the present quantitative findings regarding the DAMP proteins may refer to a cumulative effect attributed to both the intra- and extracellular level.

Moreover, we acknowledge that some selected DAMPs (fibronectin, S100A11, HSP90 and galectin-3) may be correlated with foam cell formation from macrophages which may lead to their over-regulation inside the aorta atherosclerotic plaques^34–37^.

Taken together, our experiments demonstrated that the mechanism underlying the atherosclerotic elevated aortic oxidative stress relates to the deregulated expression of proteins involved in ferroptosis and necroptosis. Based on these novel findings, we conclude that the dysregulation of specific proteins involved in non-apoptotic regulated cell death mechanisms plays a significant role in the sustained inflammatory status which recommends them as new targets in the personalized treatment of atherosclerosis.

## Methods

### Reagents

The following reagents were of LC–MS grade and provided by Merck KGaA (Darmstadt, Germany). *Complete* protease inhibitor cocktail was purchased from Roche (Mannheim, Germany). C18 solid phase extraction (SPE) columns were acquired from Waters Corporation (Milford, USA). Thiobarbituric acid reactive substances (TBARS) are provided from Merck KGaA (Darmstadt, Germany) and Evolocumab, a monoclonal antibody against proprotein convertase subtilisin/kexin type 9 (PCSK9) from Amgen Europe BV (Breda, Netherlands). The monocyte cell line THP-1 (Tib-202) was purchased from the American Type Culture Collection (ATCC, Manassas, U.S.A.).

### Animals

Twenty-nine healthy males, twelve weeks old New Zealand White rabbits were purchased from ‘Cantacuzino’ National Institute of Research and Development for Microbiology and Immunology (Bucharest, Romania) and housed in suitable facilities under controlled temperature, humidity and 12 h light cycles. The animals were randomized into four experimental groups: (C) control group (n = 7) that received standard chow diet (100 g/day) for twelve weeks; (Ae) early atherosclerotic group (n = 6) that received a high-fat diet (0.5% cholesterol and 5% corn oil), for the first eight weeks, after which they were switched to a standard diet for another four weeks; (As) stabilized atherosclerotic group (n = 8) that received a high-fat diet for the first eight weeks, followed by a standard diet combined with lipid-lowering treatment (atorvastatin and PCSK9 antibody); (Al) advanced, late atherosclerotic group (n = 8) that received a high-fat diet for twelve weeks (Fig. 1a). The lipid-lowering treatment consisted of daily oral atorvastatin gavage administration (3 mg/kg body weight daily) along with subcutaneous administration of monoclonal antibody against PCSK9 (Evolocumab, 25 mg/kg body weight weekly).

All experimental protocols were approved by the Ethic Committee of Institute of Cellular Biology and Pathology “Nicolae Simionescu” and by the National Sanitary Veterinary and Food Safety Authority (No. 365/12.07.2017) in accordance with Directive 2010/63 of European Union.

### Blood sampling and lipid measurement

Blood samples were harvested from the auricular artery after a 12 h fasting period, in Prima Vacutainer clot activator tubes (Hamburg, Germany). The serum separated by centrifugation at 1300×*g* for 10 min at room temperature, was collected and stored in small aliquots at − 80℃ until use (Fig. 1b). Total cholesterol, LDL-cholesterol, triglycerides were measured according to the protocols of the commercial kits from DIALAB GmbH (Wiener Neudorf, Austria). This study used only male rabbits to avoid bias due to gender differences in estrogen levels. Female rabbits are more susceptible to seasonal variations in estrogen levels^20^, so the main findings cannot be extrapolated to females.

At the end the rabbits were euthanized with a cocktail of ketamine hydrochloride 25 mg/kg and xylazine 5 mg/kg, for aorta collection. The tissue samples were stored at − 80 °C in a protease inhibitor cocktail for future processing.

### Aortic tissue sample preparation for protein extraction

Mechanical tissue homogenates (30 mg) from the ascending thoracic aorta was made in a lysis buffer containing 8 M urea, 1% DOC and 100 mM Tris–HCl (pH 7.5). The protein-rich supernatant was separated by centrifugation (20,000×*g*, 20 min, 4 °C). Protein BCA assay kit (Thermo Scientific) was selected for all protein extracts subsequently used for biochemical assays, mass spectrometry analysis and Western blot experiments (Fig. 1b).

### In vitro cell culture experiments

Human monocytic cells (THP-1) were seeded (5 × 10^5^ cells/ml) and cultivated for 48 h, at 37 °C and 5% CO_2_ in RPMI medium supplemented with 0.5% serum harvested from control (sC) and late atherosclerotic (sAl) rabbit groups in 3 biological replicates. After 48 h, total protein was extracted as previously described for aortic tissue for mass spectrometry analysis.

### Liquid chromatography—mass spectrometry (LC–MS/MS) analysis

Equal amounts of protein samples (50 µg) were purified by acetone precipitation, using a 1:4 volume ratio (protein sample to ice cold acetone) and incubation at − 28 °C for 2 h. The samples were suitably processed for LC–MS/MS analysis as mentioned previously^38^. The Easy-nLC II nano-chromatographic system was coupled to the LTQ Orbitrap Velos Pro hybrid mass spectrometer (both from Thermo Scientific) for the analyses. For each sample, 500 ng of peptides were separated into a 50 cm µPAC C18 microchip nano-LC column (5 µm pillar diameter, 2.5 µm inter-pillar distance, 18 µm pillar length/bed depth, 315 µm bed channel width) (PharmaFluidics). To overcome the 3 µL void volume of the column, a flow gradient was applied from 750 to 300 nL/min for the first 10 min of the chromatographic method, using a concentration gradient from 5% buffer B (0.1% FA, 99.9% ACN) to 10% buffer B. The separation consisted of a 90 min 10–35% buffer B gradient. A grounding cable was attached to the after-column union to avoid electrospray interferences and 2.3 kV were applied using the liquid junction method on the connection before the silica tip emitter (New Objective PicoTip). The mass spectrometer was operated in a Top 15 data-dependent analysis with 60 k resolution on the full 350–1700 m/z domain, while precursor fragmentation was performed using collision-induced dissociation (CID). Internal calibration was done using the 445.120028 Da polysiloxane peak.

### Mass spectrometry data analysis

The raw files were qualitatively and quantitatively analyzed using the Proteome Discoverer 2.4 software (Thermo Scientific). UniProt 9986 *Oryctolagus cuniculus* reference proteome was used for protein inference, with methionine oxidation set as a dynamic modification and cysteine carbamidomethylation as a static one, while a maximum of two missed cleavages was allowed. A reverse database searching was performed for strict protein and peptide FDR settings (< 1%). Label-free relative quantification was performed on the precursor intensity level, with 90% of replicate features and ANOVA hypothesis test enabled. Normalization was engaged in the total peptide amount on controls average. Proteins with a SequestHT score > 10, quantified based on at least two peptides, that were significantly differentially abundant amongst the compared groups (> 1.5-fold ratio and *p* value < 0.05) were retained for further analysis. STRING database algorithm (v. 11.0) was applied for computational prediction of protein–protein interaction based on genomic context, high-throughput lab experiments, automated text mining and accumulated database knowledge. STRING was also used for gene ontology and KEGG signaling pathways over-representation.

### Histological studies

OCT embedded aortic segments were rapidly frozen in liquid nitrogen and serially sectioned and stained with Oil Red O for lipid deposits (red) and Hematoxylin for cell nuclei (blue). Prussian Blue and Nuclear Fast Red solution was used for ferrous and ferric iron blue, cytoplasm pink and cell nuclei in red detection. The sections were mounted in 90% glycerol and histological images were captured using an AxioVert.A1 inverted microscope (Carl Zeiss GmbH, Göttingen, Germany) and analyzed with Zen Pro 2012 Software (Carl Zeiss, GmbH).

### Western blot assays

Western blot immunoassays were performed as previously^38^. SDS-PAGE separated proteins (50 μg protein/lane), were transferred onto nitrocellulose membranes (Bio-Rad Laboratories, Germany) and incubated with the following primary antibodies (dilution 1:1000): Anti-Malondialdehyde (Ab27642,), Anti-4 Hydroxynonenal (Ab46545), Anti- Voltage-dependent anion-selective channel protein 3 (TA38558), Anti-Protein S100A11 (PA5-21330), Anti-Calreticulin (PA3-900), Anti-Fibronectin (Ab6328) and Anti- β-Actin (Ab6276). The appropriate horseradish peroxidase (HRP)-conjugated secondary antibody was used (dilution 1:2000). In some instances, physical cropping of blot membranes was performed prior to blocking and incubating with primary antibodies, in order to perform multiple parallel antigen analyses from the same membrane blot. The immune complexes were detected by enhanced chemiluminescence using a digital detection system (ImageQuant LAS 4000, GE Healthcare).

### Gene expression analysis

Total RNA was extracted from aorta tissue using the RNeasy Mini Kit (QIAGEN, Hilden, Germany). The nucleic acid quality was assessed using an Agilent 2100 Bioanalyzer (Agilent Technologies, Santa Clara, CA) and quantified by NanoDrop ND 1000 spectrophotometer (Thermo Scientific). The cDNA was generated from 1 μg total RNA using Transcriptor First Strand cDNA Synthesis Kit (Roche, Mannheim, Germany). LightCycler 480 SYBR Green I Master mix was used to perform qPCR in the LightCycler System (Roche). All reactions were performed in triplicate, and the product specificity was validated by melting-curve analysis. Amplification of the housekeeping gene β-Actin was used for normalization. The primer sequences selected are provided as supplementary information (Supplementary S1). Relative quantification of gene expression was performed with LightCycler 480 Software using the Ε (Efficiency) method.

### Assessment of serum lipid peroxidation

Quantitative serum lipid peroxidation assay was performed to evaluate the concentrations of thiobarbituric acid reactive substances (TBARS) as an indicative of lipid oxidative stress. The concentrations of TBARS were calculated using malondialdehyde (MDA) as a reference standard. Briefly, 200 µl serum was mixed with 200 µL ice cold 10% trichloroacetic acid to precipitate proteins. After 15 min on ice, the samples were centrifuged at 2200×*g* for 15 min at 4 °C. Standards and 200 µL supernatant mixed with an equal volume of 0.67% (w/v) TBA were incubated in a boiling water bath for 10 min. After cooling to room temperature, the reaction mixture was centrifuged at 4000×*g* for 10 min and the absorbance of the supernatant was measured at 532 nm using the same PHERAstar FS system.

### Statistics

The results were expressed as the mean ± standard deviation of at least six experiments in two technical replicates. Statistical analyses were performed using GraphPad Prism 5.0 software using t Student test analyses to obtain statistical significance (*p < 0.05, **p < 0.01, ***p < 0.005). For mass spectrometric bioinformatics analysis, statistical algorithms (Benjamini–Hochberg procedure) were employed for correcting the *p *values based on the false discovery rate (FDR).

## Supplementary Information

Supplementary Information 1.

Supplementary Information 2.

Supplementary Information 3.

## Data Availability

The Mass spectrometry data were deposited in the PRIDE^39–41^ repository via ProteomeXchange with the dataset identifier PXD026379. We confirm that our study is reported in accordance with ARRIVE guidelines.
