# Supplementary material for: Regulated cell death joins in atherosclerotic plaque silent progression
Source: Sci Rep. 2022 Feb 18;12:2814. doi: 10.1038/s41598-022-06762-y (PMC8857202; doi:10.1038/s41598-022-06762-y)
Supplement: Supplementary file 1 — Supplementary Information 1. [file 41598_2022_6762_MOESM1_ESM.docx]

**Supplementary information S1**

**Regulated cell death joins in atherosclerotic plaque silent progression**

*Elena Uyy,^1^ Viorel I. Suica,^1^ Raluca M. Boteanu,^1^ Aurel Cerveanu-Hogas,^1^ Luminita Ivan,^1^ Rune Hansen,^2,3^ Felicia Antohe^1*^*

^1^Department of Proteomics, Institute of Cellular Biology and Pathology “Nicolae Simionescu” of the Romanian Academy, Bucharest, Romania

^2^Department of Health Research, SINTEF Digital, Trondheim, Norway

^3^Department of Circulation and Medical Imaging, Norwegian University of Science and Technology, Trondheim, Norway

**Corresponding Author information:**

*Corresponding Author: Felicia Antohe

Postal address: Proteomics Department, Institute of Cellular Biology and Pathology “Nicolae Simionescu”, 8, B.P. Hasdeu Street, PO Box 35–14, 050568, Bucharest, Romania;

Phone: +40 21 3192737;

Email: [felicia.antohe@icbp.ro](mailto:felicia.antohe@icbp.ro)

Western blot assay Supplementary Information for Figure 2.


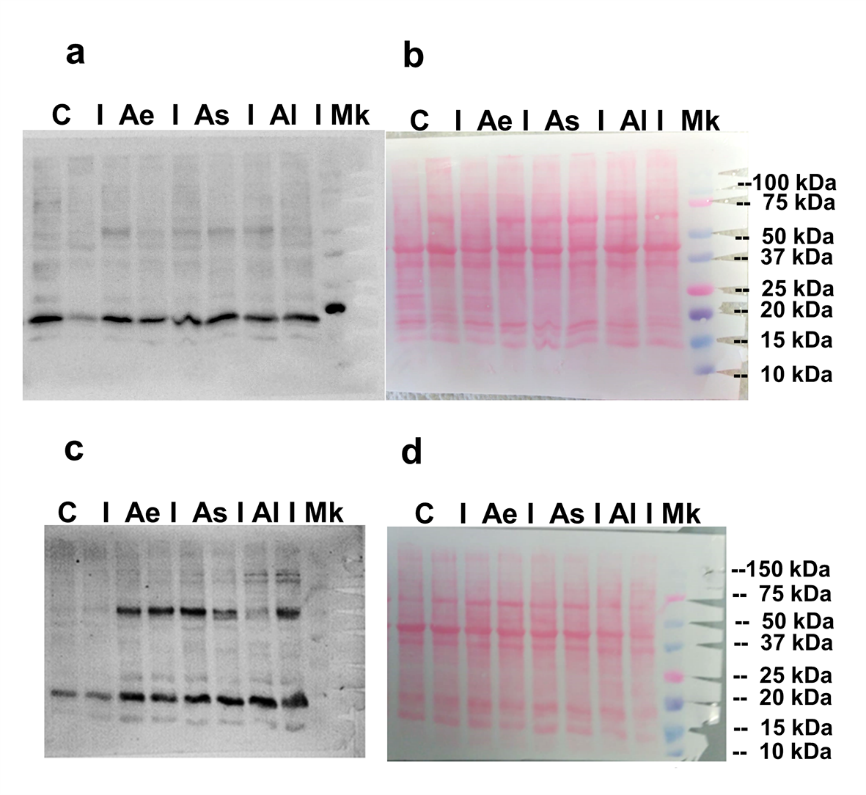


**Figure 1.** Full-length blots of the images presented in Figure 2, which were used for the detection of (a) malondialdehyde MDA- and (c) hydroxynonenal HNE- proteins in the ascending aorta’s homogenate. We stained the membranes with (b, d) Ponceau S to detect the protein signal prior to blocking and incubating with the primary antibodies, which was also used for normalization of the MDA- and HNE-proteins’ level.

Western blot assay Supplementary Information for Figure 6.


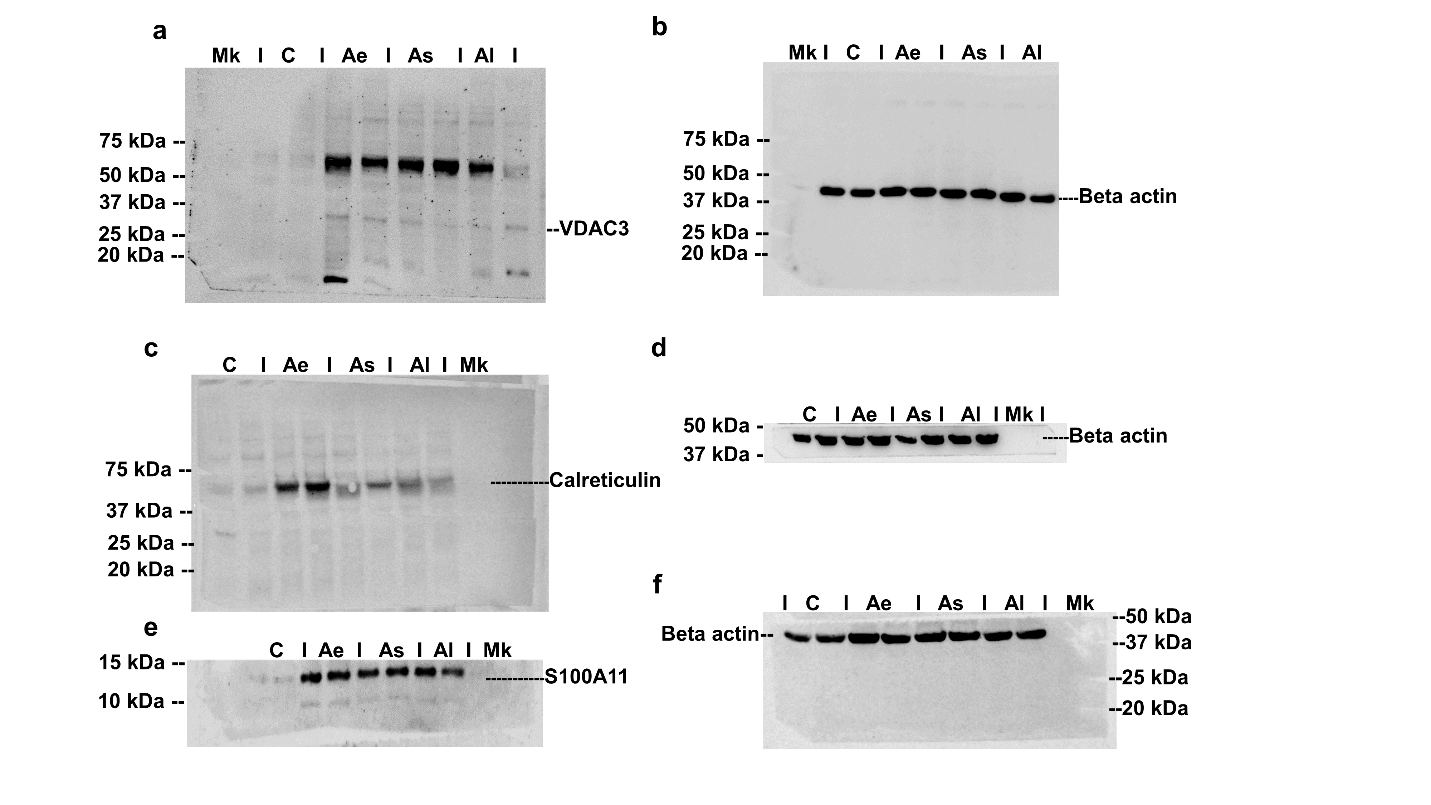


**Figure 2.** The full uncropped nitrocellulose images used to evidence the positive reactions from Figure 6 for a) VDAC3, b) beta actin for VDAC3 normalization, c) calreticulin, d) beta actin for calreticulin normalization, e) S100A11 and f) beta actin for S100A11 normalization. We stained the membranes with Ponceau S to detect the protein signal. Afterwards, the membranes were cut in sections specific to the antigens of interest, prior to blocking and incubating with primary antibodies, for technical reasons (use of smaller volume of antibody solution; possibility to perform multiple parallel incubations from the same full membrane).

**Table 1:** The primer sequences used in qPCR experiments.

| GENE  (gene symbol) | Forward primers | Reverse primers |
| --- | --- | --- |
| Ferritin light chain (FTL) | 5′-ACCTACCTCTCTCTGGGCTTC-3' | 5′-GTCTTGCCCCACTCATCCTG-3′ |
| Voltage-dependent anion channels (VDAC3) | 5′-GGCTGCCAAGGATGTCTTCA-3′ | 5′-GGTCTCTAGGTTGCCTGACG-3′ |
| Transferrin (TF) | 5′-GACCTTCGGGGCAAAAACAC-3′ | 5′-TGGCACAACACTCTCAAGCA-3′ |
| Tool like receptor 4 (TLR4) | 5′-GAAATCTGGGAGCCCTGTGTG-3′ | 5′-GCTATGGCTGCCTAAATGCTC-3′ |
| Tool like receptor 2 (TLR2) | 5′-CCTGCTGACGCTGAAAAACC-3′ | 5′-TCAGCCGTCTCAACCTTTCC-3′ |
| Calreticulin (CALR) | 5′-AAGGAGCAGTTTCTGGACGG-3′ | 5′-GAACTTGCCCGAACTGAGGA-3′ |
| β-Actin | 5′-GTGCTTCTAGGCGGACTGTT-3′ | 5′-CGGCCACATTGCAGAACTTT-3′ |
